# Trauma, a Matter of the Heart—Molecular Mechanism of Post-Traumatic Cardiac Dysfunction

**DOI:** 10.3390/ijms22020737

**Published:** 2021-01-13

**Authors:** Birte Weber, Ina Lackner, Florian Gebhard, Theodore Miclau, Miriam Kalbitz

**Affiliations:** 1 Department of Traumatology, Hand-, Plastic-, and Reconstructive Surgery, Center of Surgery, University of Ulm, 86081 Ulm, Germany; birte.weber@uni-ulm.de (B.W.); ina.lackner@uni-ulm.de (I.L.); florian.gebhard@uniklinik-ulm.de (F.G.); 2 Orthopaedic Trauma Institute, Department of Orthopaedic Surgery, University of California, 2550 23rd Street, San Francisco, CA 94110, USA; Theodore.Miclau@ucsf.edu

**Keywords:** post-traumatic cardiomyopathy, multiple trauma, markers of cardiac damage, complement system, extracellular histones, inflammation, complement activation, TBI, stress-related cardiomyopathy, burn injury

## Abstract

Trauma remains a leading global cause of mortality, particularly in the young population. In the United States, approximately 30,000 patients with blunt cardiac trauma were recorded annually. Cardiac damage is a predictor for poor outcome after multiple trauma, with a poor prognosis and prolonged in-hospitalization. Systemic elevation of cardiac troponins was correlated with survival, injury severity score, and catecholamine consumption of patients after multiple trauma. The clinical features of the so-called “commotio cordis” are dysrhythmias, including ventricular fibrillation and sudden cardiac arrest as well as wall motion disorders. In trauma patients with inappropriate hypotension and inadequate response to fluid resuscitation, cardiac injury should be considered. Therefore, a combination of echocardiography (ECG) measurements, echocardiography, and systemic appearance of cardiomyocyte damage markers such as troponin appears to be an appropriate diagnostic approach to detect cardiac dysfunction after trauma. However, the mechanisms of post-traumatic cardiac dysfunction are still actively being investigated. This review aims to discuss cardiac damage following trauma, focusing on mechanisms of post-traumatic cardiac dysfunction associated with inflammation and complement activation. Herein, a causal relationship of cardiac dysfunction to traumatic brain injury, blunt chest trauma, multiple trauma, burn injury, psychosocial stress, fracture, and hemorrhagic shock are illustrated and therapeutic options are discussed.

## 1. Introduction

Trauma remains a leading cause of mortality in the developed world. Blunt chest trauma is recorded in 45% of multiply injured patients [1]. In addition to lung injury, cardiac damage was identified as a predictor for a poor outcome after trauma [2] and is, therefore, considered in the abbreviated injury scale (AIS) and consequently in the injury severity score (ISS) [3]. Another useful tool for assessing the severity of cardiac injury may be the American Association for the Surgery of Trauma heart injury scale. In general, cardiac injuries are associated with dysrhythmias, ventricular fibrillation, impaired cardiac function, and sudden cardiac arrest [4,5]. Moreover, heart injuries following multiple trauma are characterized by a prolonged ventilation interval as well as by a longer hospital stay of the patients [6]. Accordingly, 78% of trauma patients with an impaired left ventricular stroke work index died after blunt chest trauma [2]. The incidence rate of blunt chest trauma varies greatly in the literature between 8% and 86%. Additionally, the myocardial damage presents a wide range from asymptomatic to death based on the severity and mechanism of the injury. Cardiac manifestations include arrhythmias, wall motion abnormalities, myocardial wall rupture, and valve damage, although the most prevalent pathology is myocardial contusion. Therefore, the diagnostic of cardiac trauma is difficult.

In addition to the standard trauma diagnostic and examination, the diagnostic algorithm for cardiac injury includes electrocardiogram (ECG) and measurement of systemic troponin (Tn) concentrations based on the guidelines of the ATLS. At first glance, this diagnostic approach seems to be appropriate, because the appearance of atrial fibrillation (AF) was observed in 5% of patients after trauma on intensive care unit (ICU) and has been associated with the ISS, the need for fluid resuscitation and catecholamines, as well as the development of systemic inflammatory response syndrome (SIRS), sepsis, and a two-fold increased mortality compared to ICU-patients without AF [7]. Nevertheless, when there is a clinical suspicion of a cardiac trauma, further diagnostics (echocardiography or CT scans) might be necessary. A high index of suspicion is required in the evaluation of a blunt injury; this early suspicion is only effective when combined with comprehensive methods of diagnosis and management (Figure S1). The investigation begins with classic methods such as vital sign monitoring, serial ECG, serial cardiac enzyme testing, chest X-ray, and FAST. The AAST has adopted the guidelines for the evaluation and treatment of myocardial contusion established by the Eastern Association for the Surgery of Trauma; recommends the use of admission ECG and troponin I in all patients, in whom BCI is suspected which can be ruled out only if both ECG result and troponin I level are normal. Echocardiogram is not effective as a screening tool for BCI and should be reserved for patients with hypotension and/or arrhythmias. Beta-blocker therapy improved survival in patients with atrial arrythmias after trauma [7]. Accordingly, risk factors for trauma-associated arrhythmias are ISS, SIRS/sepsis, the need for catecholamines, and furthermore age, a simplified acute physiology score > 30, female sex, and head injury [8]. Furthermore, after multiple trauma, increased serum troponin levels were associated with increased mortality and are correlated with the ISS [9].

This review aims to provide an overview of cardiac trauma associated with both direct cardiac injury and as secondary damage due to the systemic inflammation.

## 2. Review

### 2.1. Troponin and Heart Fatty Acid Binding Protein (HFABP): Markers for Post-Traumatic Cardiac Injury

Troponins (T; C; I) are small proteins that play an important role in the calcium-triggered contraction of the heart muscle. In critically ill patients, elevated cardiac troponin T levels were associated with in-hospital mortality, but did not correlate with differences in long-term survival [10]. Furthermore, elevated troponin T levels in ICU patients after multiple trauma were correlated with the ISS score, AIS, survival, and the need for catecholamines [9]. Following trauma, a systemic elevation of troponin has been associated with cardiac contusion, occurring in 15–45%. In post-traumatic patients, a systemic troponin elevation was described as a sensitive biomarker for the detection of cardiac-related complications, particularly in combination with echocardiography [11,12]. In recently published reports, troponin elevation was observed in different experimental trauma models and different species, including after multiple trauma in mice [13], asphyxia and hemorrhage in newborn pigs [14] and in multiple trauma with hemorrhagic shock in pigs [15]. One limitation of troponin in the trauma context, particularly after multiple trauma and in old patients, is the kidney function: a 50% reduction of kidney clearance is associated with a two-fold increase in cardiac TnT [16].

Before leaky cell membranes of CMs were found to contribute the troponin release, cell necrosis had been postulated as the exclusive mechanism for the troponin release [17]. In addition to necrosis and apoptosis, there is increasing evidence for diverse possible mechanisms for the reversible systemic increase of troponin [17]. This reversible cardiomyocyte (CM) injury has been related to the release of microparticles, membrane blebs, and a higher permeability in the sense of a cell wound [18,19,20,21], as well as a response to distension [22]. It is expected that fragmented cardiac troponin originates from irreversibly damaged CMs, whereas the appearance of systemic intact troponins results from reversible injury [23]. The structurally bound troponin is degraded by calpains, which react to increases in intracellular calcium or pH shifts [17]. It is well known that different danger associated molecular patterns (DAMPs), including extracellular histones, induce an elevated in intracellular calcium in CM via increased permeability of cell membrane or reactive oxygen species (ROS) production [24]. Caspase-3 has been described to cleave cardiac troponin T complexed with troponin I and C, but not free cytosolic troponin [25]. Additionally, Hessel et al. (2008) described the appearance of intact troponin as unlikely in a model of irreversible cell damage, whereas both the intact and fragmented forms were observed in a model of definitive irreversible CM cell [22,26]. Furthermore, there is evidence that troponin I itself reacts on the heart; troponin has been shown to provoke inflammatory heart disease in mice [27].

Another biomarker of myocardial damage is the HFABP, which appears earlier in the circulation after myocardial infarction relative to troponin [28]. HFABP is the most sensitive marker for early damage of CM, but appears to be less specific than troponin, given its appearance in skeletal muscle [29]. A systemic increase of HFABP was recently identified early after experimental multiple trauma in pigs [15]. HFABP has recently been used clinically in the diagnosis of cardiac damage. Therefore, the available literature is limited. In the future, clinical studies in humans must show whether this marker is also suitable for the diagnosis of cardiac damage after trauma.

### 2.2. Functional Impairment after Traumatic Heart Injury

In pigs after multiple trauma, a reversible reduction of the ejection fraction and shortening fraction was described [15]. In addition to these systolic changes, the diastolic function might also be influenced by trauma. Further studies are necessary to evaluate the systolic and diastolic functions by echocardiographic measurements after trauma.

In addition to systolic and diastolic dysfunction, arrhythmias have been described after trauma. Analysis of all trauma patients from an American College of Surgeon verified Level I Trauma center found 258 trauma patients over two years diagnosed with new-onset atrial fibrillation (AF) after trauma [30]. In the literature about commotio cordis, surprisingly, there are many reported cases of sudden cardiac death induced by a baseball blow against the chest [31]. The blunt chest blow triggers ventricular fibrillation, which can lead to sudden cardiac death on the field [32]. The frequency of commotio cordis via chest blows by soccer balls appears to contradict the prevailing notion that air-filled projectiles convey less risk for ventricular fibrillation than balls with a solid core such as baseballs or lacrosse balls [32]. The development of ventricular fibrillation after baseball-induced commotio cordis in pigs was related to the peak left ventricular pressure produced by the blow. These changes in the left ventricular pressure were associated with cell membrane stretch and mechanical activation of ion channels [33]. In addition to a direct impact on the heart, there are also reports of patients developing arrythmias after trauma without any effect on the chest. For example, one case described the development of QTc prolongation after a mild concussion in a pediatric patient [34].

In addition to impaired systolic and diastolic function and arrhythmias, heart valve insufficiency has been observed after trauma. Traumatic rupture of the chordae tendineae was described to lead to acute severe mitral regurgitation following motor vehicle accidents [35]. Furthermore, different case reports measured trauma-associated severe tricuspid regurgitation [36,37]. The few published case reports of traumatic valvular damage showed a wide variation of symptoms; patients can be asymptomatic for years or hemodynamically unstable directly after trauma. Most of the patients developed symptoms within the first seven days [38,39]. Valvular lesions resulted from high-energy direct trauma on the chest, including car accidents and falls from heights. The most probable mechanism is the sudden deceleration or compression of the blood column in the heart during a vulnerable valve phase [38]. The most susceptible valves appear to be the atrioventricular valves [40]. In summary, the topic of traumatic valvular lesions might be underestimated in recent research on cardiac contusions, because of its wide variation in symptoms and difficult diagnosis.

#### 2.2.1. Inflammation

Elevated cytokine levels, including tumor necrosis factor (TNF), interleukin (IL)-1β, and IL-6 have been described to be cardio-depressive [41]. Following multiple trauma in pigs, IL-1β and IL-6 were elevated in cardiac tissue of the left ventricle [15], while IL-6 was also increased systemically after multiple trauma [42]. Early elevated IL-6 levels in mice were associated with a poor outcome after sepsis [43]. An increase of the systemic IL-6 concentration has been related to cardiac dysfunction, demonstrated as a reduction of the stroke volume, cardiac output, and performance of the left ventricle [44,45]. In rats with hemorrhagic shock, treatment with an anti-IL-6 antibody preserved the reduction of cardiac output and decreased the nuclear factor ‘kappa-light-chain-enhancer’ of activated B-cells (NFkB), intercellular adhesion molecule 1, myeloperoxidase (MPO), and cytokine-induced neutrophil chemoattractant 1 and 3 activities [45]. In the presence of plasma from mice with mechanical trauma, apoptosis has been increased in CMs (peak after 12 h). Here, increased apoptosis was reduced by the application of anti-TNF-antibody etanercept. [46]. Furthermore, cardiac MPO activity was elevated early after blunt chest trauma in rats, which might be associated with the formation of oxygen- and nitrogenous radicals [47]. Cytokines are described to lead to activation of inducible nitric oxide synthase (iNOS) in the context of septic cardiomyopathy [48]. High iNOS activity can contribute to nitrosative stress in the heart, which is detectable by nitrotyrosine staining. Recently, nitrotyrosine elevation was demonstrated in the heart after experimental multiple trauma in a pig model [15]. In cultured Cm, the presence of IL-6 led to a time-dependent increase of iNOS protein. Furthermore, this study presented a decrease in intracellular calcium due to IL-6, which could have led to a change in the CM contractibility [49]. In rat CMs, the presence of TNF and IL-1β led to dysfunction of the calcium balance, prolonging the transient duration of calcium, and therefore, the action potential, and leading to asynchronous calcium release during electrical stimulation. Furthermore, these cytokines increased the vulnerability of the sarcoplasmic reticulum for spontaneous calcium leakage. TNF and IL-1β depressed transient calcium and contractility, therefore leading to arrhythmogenicity in ventricular rat CM [50]. The presence of IL-1β may be associated with prolonged action potential duration and the reduction in the transient potassium current of 35%, thereby reducing repolarization in the CM and increasing diastolic sarcoplasmic calcium leakage. Together, these changes led to a high potential for cardiac arrythmias [51]. In the case of viral myocarditis in mice, high levels of local TNF were described to activate NFkB signaling, leading to the inhibition of constituents of fatty acid β-oxidation, oxidative phosphorylation, and peroxisome proliferator-activated receptor gamma coactivator 1 signaling. Therefore, TNF might be responsible for changes in myocardial energy metabolism during post-traumatic cardiomyopathy [52]. Taken together, for proinflammatory mediators like TNF and IL-1β manifold mechanisms were described as leading to CMs dysfunction and proarrhythmogenic potential.

#### 2.2.2. Systemic Release of DAMPs and the Impact on the Heart

To an early systemic inflammatory response, accompanied by the systemic release of DAMPs [53], in humans, high-mobility-group-protein B1 (HMGB-1) release was described 30 min after trauma [54] and was associated with injury severity, complement system activation, and mortality [54]. Additionally, in an experimental setting of multiple trauma, including a chest trauma with hemorrhagic shock in pigs, HMGB-1-elevation was described [55]. HMGB-1 is known to induce CM dysfunction, for example, in cardiac hypertrophy and heart failure [56], in inflammatory heart disease [27], and in myocardial ischemia/reperfusion injury [57]. HMGB-1 also acts as a second DAMP, the extracellular histones, via toll-like receptors (TLR), particularly TLR-2, -4, and -9. Extracellular histones were associated with trauma-induced lung injury and acute respiratory distress syndrome in humans [58,59] as well as septic cardiomyopathy in mice [24]. DAMPs can lead to an increased intracellular calcium concentration in CMs, which has been related to the appearance of bradycardia and bigeminy [24]. In patients with sepsis, circulating histones were elevated and correlated with increased troponin levels, the need for noradrenalin-application, and the appearance of left ventricular dysfunction and arrythmias [60]. In experimental blunt chest trauma in rats and multiple trauma in pigs and mice, we observed systemic release of circulating histones [13,15,47]. Following the systemic application of extracellular histones in mice, an increase of inflammatory cytokines, including TNF, IL-1β, IL-6, and IL-10 was observed [61]. Furthermore, HMGB-1 was associated with the production of inflammatory cytokines [62,63], including TNF, IL-1β, and IL-6 [64]. Circulating histones accumulate in the heart and were associated with defective CM function, as well as dose- and time-dependent ROS production and increase of intracellular calcium [24]. Histones also reduced the mitochondrial membrane potential and the ATP production in a dose-dependent manner, resulting in reduced CM contraction due to a lack of energy [24,65]. TLR-4 has been found to play an important role in cardiac dysfunction after trauma; its absence ameliorated cardiac dysfunction in mice during trauma hemorrhagic shock [66]. In humans after trauma left ventricular dysfunction was observed [67].

Recently, another damaging molecule for the heart after trauma was described, the inflammatory cytokine and heparin-binding growth- and differentiation factor midkine [68]. Systemic midkine elevation was observed after bone fracture, in burn injury, and after traumatic spinal cord injury [69,70,71]. When released after fracture in patients, it has an ongoing elevation and remains elevated over a period of 42 days [70]. In pigs with multiple trauma, including chest trauma, a systemic increase of midkine was measured [68]. The molecular effects of midkine on CM are strongly debated, with reports of improved cardiac function, enhanced angiogenesis, and reduced cardiac remodeling in the context of midkine presence, as well as reports of decreased cell survival, fibrosis, and CM hypertrophy due to midkine in ischemic/reperfusion models [72,73,74,75,76]. Our group recently described a different mechanism of the damaging effects of midkine on human CMs. In this context, midkine led to massive changes in calcium handling of the cells, as observed through increases in delta calcium peaks, decreases of frequency in calcium peaks, and enhancements of mRNA production of the calcium-handling-proteins Sarcoplasmic/endoplasmic reticulum calcium ATPase (SERCA) and Na^+^/Ca^2+^ exchanger [68]. In addition to influencing the contractility via intracellular calcium alterations, midkine also decreased the mitochondrial function of Cm and led to their apoptosis [68].

Taken together, the molecular mechanisms for DAMP-associated cardiac dysfunction are associated with changes in the calcium homeostasis or calcium handling proteins, mitochondrial function, ATP production, and regulation of local inflammatory cytokine release.

#### 2.2.3. Complement Activation after Trauma and the Impact on the Heart

During experimental sepsis and after burn injury, the complement activation product complement factor (C5a) has been found to induce dramatic contractile dysfunction in CMs in vitro and in vivo by interaction with C5a receptors [65,77,78]. C5a receptor (C5aR) knockout mice presented significantly lower TNF expression levels after ischemic trauma, but no changes were observed in IL-6 or IL-1β [79]. Furthermore, expression of matrix metalloprotease (MMP9) and junction adhesion molecule-A, molecules that are involved in leukocyte transmigration, were reduced in C5aR-knockout mice. This led to reduced infarct size and diminished leukocyte recruitment in the heart [79]. In trauma patients, activation of the complement system, particularly an increase of the activated C5a, was observed [80]. Therefore, high systemic levels of C3a and C5a were correlated with the severity of the trauma and were predictive for the development of acute respiratory distress syndrome and multiorgan failure [80,81,82,83,84]. In contrast to a threefold increase in myocardial C5aR expression after ischemia in experimental blunt chest trauma in mice [47], in experimental asphyxia and hemorrhage in piglets [14] and after experimental multiple trauma in pigs, complement factor C5a receptor 1 (C5aR1) expression in left ventricles was decreased [15]. Interestingly, in burn injury and after cecal ligation and puncture (CLP) sepsis, C5aR1 was also upregulated in heart tissue, which is associated with depressed cardiac function [65,77]. The reduction of C5aR1 after trauma might be due an internalization of the receptor after binding of C5a, which has been described to be significantly elevated after trauma in animal models [85,86]. Accordingly, we demonstrated a systemic consumption of factors of both the classical and alternative complement systems as measured by CH-50 in pigs 6 h after multiple trauma [87]. Furthermore, neutrophils were observed to migrate into the heart tissue after trauma, which has been shown in experimental blunt chest trauma in mice [47]. Neutrophil serine protease cleaves C5aR1 and, therefore, a reduction thereof can be measured after trauma [88]. Our own unpublished data showed that the C5aR2 was not altered in heart tissue after experimental multiple trauma in pigs [15]. Ward et al. demonstrated in 2008 that C5aR2 is not internalized after interaction with C5a, which might at least in part be the reason why C5aR2 remains unaltered in the heart after trauma [89]. Following sepsis, blockade or absence of either C5aR1 or C5aR2 significantly improved survival in animals [90]. Cardiac output and the left ventricular stroke volume were higher in C5aR2-knockout mice compared to wildtype mice after CLP [65]. Furthermore, during the inflammatory condition of CLP sepsis, C5a-C5aR1 interactions were shown to induce an excessive amount of cytosolic ROS and Ca2+i in CMs [65,91].

Mechanistically, dysfunction of the heart related to C5a was shown to be mediated by alteration of calcium-handling proteins, which again was ameliorated in the absence of either C5aR1 or C5aR2 [65]. Additionally, C5a-C5aR interactions were shown to be associated with ROS elevation in CM that was related to cardiac NLRP3 inflammasome activation and MAP/Akt phosphorylation [65,92,93]. C5a further reduced the resting membrane potential, increased intracellular calcium, and impaired calcium handling, which are potentially responsible for changes in the heart rhythm [24,65]. Additionally, calcium-handling C5a also influenced mitochondrial function and induced mitochondrial stress, which might affect the ATP availability [94]. Furthermore, a redox imbalance because of C5a and C3a presence was observed and might be induced by NADPH oxidases NOX1 and NOX2 [95]. Therefore, C5a was shown to disturb calcium balance and electrophysiological function of CM, thereby, inducing defects in heart contractility and relaxation [65,96,97].

The anaphylatoxin C3a was also shown to be responsible for cardiac dysfunction, arrythmia, and contractile failure of the heart [98]. An increase of the C3aR on the surface of CMs was associated with cardiac inflammation and progressive heart failure [99]. In the context of post-traumatic cardiac dysfunction, it is interesting that C3aR expression also correlates with the invasiveness of the chosen fracture stabilization [100]. In addition to an increase in inflammation, C3a also leads to enhanced apoptosis, which might be mediated due to the carboxypeptidase B1(CpB1)-C3-C3aR pathway, resulting in an increase of caspase 11 [101]. Taken together, the activation of the complement system has to be considered as an important mechanism for cardiac dysfunction after trauma.

#### 2.2.4. Structural Alterations

The gap junction profile of the heart plays an important role in the ion homeostasis, development of arrythmias, and ability of the heart to contract. Alterations of connexin 43 (Cx43) were found to be associated with both ischemic [102] and nonischemic [103,104] cardiac injury. In separate studies, we detected a change in the contribution to Cx43 after trauma. The strictly ordered Cx43 molecules were internalized after chest trauma, multiple trauma, asphyxia, and, in mice, social stress [14,15,47,105]. The Cx43 endocytosis was shown to be associated with changes in the spread of electrical activation and associated with arrythmia and cardiac dysfunction [106,107]. Cell–cell communication through gap junctions such as Cx43 has been shown to partly prevent apoptosis in vitro [108]. Furthermore, these alterations in Connexin distribution were associated with a colocalization of zonula occludens-1 (ZO-1) [15,109]. Moreover, gap junction alterations in structural and z-disc-associated molecules are described. The z-disc is a type of responder to stretch and mechanical tension, being necessary for the adaption to hemodynamic demands in the heart [110]. Following multiple trauma, we detected reduced levels of alpha-actinin and increased levels of the intermediate filament desmin in the left ventricle [15]. A loss of alpha-actinin might be responsible for cardiac dysfunction, because of the inability to adapt to the new hemodynamic demands. Alpha-actinin is associated with L-type calcium channels [110]. Moreover, the increase of desmin is well-known in the literature describing desminopathies, which alter heart biomechanics and calcium homeostasis [111]. Mutations in the desmin gene have been related to cardiomyopathy in up to 50% and to cardiac conduction disease or arrythmia in nearly 60% of patients [112]. In the literature, desminopathies are associated with changes in calcium amplitude of CM as well as the distribution and function of the ryanodine receptor [111]. In this context, inflammation, such as a high TNF, has been shown to induce desmin clevage by caspase-6, leading to a loss of desmin localization at intercalated discs and aggregation in CMs [113]. Gard et al. (2005), described a remodeling of gap junctions and, amongst other things, a mislocalization of Cx43 in a mouse model of desmin-related cardiomyopathy [114]. Additionally, in mice with diastolic dysfunction and guinea pigs with heart failure, an increase of desmin was reported [115,116].

#### 2.2.5. Cardiac Metabolism after Trauma

Not only have inflammation and structural alterations been reported in the context of trauma, but also in the disturbance of cardiac energy metabolism linked to trauma [117]. Following trauma, stress leads to a mobilization of endogenous substrates via catabolic hormones (glucagon, catecholamines, and cortisol), with the delivery of substrates to the brain and heart taking particular priority [118]. The systemic appearance of proinflammatory mediators, like IL-1β, IL-6, and TNF, exacerbated the bodily catabolism [119]. The heart is capable of using all classes of energetic substrates, which is a major advantage by providing constant ATP production for effective heart function [120,121,122,123]. In sepsis and ischemia, increased glycogen amount and a high glucose uptake level via the glucose transporter glucose transporter (GLUT) 4 in the heart were described [124]. This shift from long-chain free fatty acids to glucose supports heart function. By contrast, after trauma, an increase in PAS-stained glycogen in the left ventricle was observed, accompanied by downregulation of the GLUT 4 transporter on CMs [15]. This decreased GLUT 4 expression on CMs after trauma is in contrast to previously published findings in ischemic diseases, where GLUT 4 was found to be translocated from intracellular to the plasma membrane [125]. Our results in asphyxiated piglets with hemorrhagic shock and cardiopulmonary resuscitation were in accordance with these results [14]. GLUT 4 transporter protein expression in the left ventricles of neonatal piglets following asphyxia and hemorrhage was increased after 6 h, whereas the cardiac glycogen concentration was reduced. The presence of GLUT 4 was shown to be associated with cardiac function; ischemic mice with GLUT 4 deletion displayed reduced systolic and diastolic function of the heart [126]. Additionally, the presence of glucose and fatty acid transporters appears to depend on the severity/mechanism of the trauma. In pigs with a unilateral femur fracture, the HFABP and CD36 (both fatty acid transporters) mRNA concentration were decreased, whereas in pigs with multiple trauma (a unilateral femur fracture and chest trauma), HFABP was increased in the left ventricle [117]. In vitro incubation of human CM with a proinflammatory polytrauma cocktail led to increased GLUT 1 mRNA and, at the same time, decreased fatty acid transporters HFABP and CD36 mRNA [117]. In addition to these observations, in vitro studies revealed that the presence of DAMPs including extracellular histones and the heparin binding growth factor midkine, was associated with alterations of the basal and spare respiratory rates of mitochondria in human CMs [68]. Further investigations related to heart glucose and fatty acid metabolism are necessary to understand the changes in cardiac metabolism after trauma and thus find ways to improve patient outcomes by providing the requisite energy supply.

### 2.3. Secondary/Indirect Cardiac Damage

An excellent example of indirect cardiac damage after trauma is burn injury. During the first 48 h after burn injury, patients suffer depressed heart contractility and output, a so-called ebb phase after burn trauma [127]. This state, characterized by a phase of tachycardia, high oxygen demand, high catecholamine levels and hyperinflammation, lasts [128,129] up to one-year after burn [130]. The high levels of catecholamines were postulated to lead to the uncoupling of β-adrenergic receptors from G-proteins, which has significant consequences for the heart. For example, calcium homeostasis is impaired, with SERCA levels and ryanodine receptor expression significantly reduced [131]. Furthermore, the activation of the β-adrenergic receptor is associated with the activation of NFkB and p38 mitogen-activated protein kinase (MAPK) which in turn regulates TNF and IL-1β release [130]. Following burn injury, proinflammatory components, including TNF, activate iNOS. Nitric oxide (NO) is cytotoxic and contributes to ventricular dysfunction [132]. Additionally, apoptosis and caspase-3 activity were described after experimental burn injury in rats, which were induced by high calcium levels and have been related to phosphoinositide 3-kinase/protein kinase B (Akt), p38 MAPK, and extracellular signal-regulated kinase pathways [130,133].

Another clinically relevant scenario of indirect cardiac dysfunction after trauma is traumatic brain injury (TBI). TBI remains the leading cause of mortality in trauma patients [134]. Interestingly, after TBI, the appearance of neurogenic pulmonary edema has been described, which has been associated with cardiac dysfunction and increased pulmonary vascular pressure as measured by echocardiography, pulmonary artery catheterization, or post-mortem biopsy [135]. Isolated TBI was associated with cardiac dysfunction (12%, with a left ventricular ejection fraction below 50%) and increased in-hospital mortality in 20% [136]. Other patients with isolated TBI presented with elevated troponin I levels and abnormal ECHO findings [136]. The association between neurological injury and cardiac dysfunction has been described in different experimental models of brain injury, including psychological stress, ischemia, and intracranial hemorrhage [137,138,139]. The brain–heart interaction based on catecholamines release, parasympathetic dysfunction, and uncontrolled inflammation, can lead to myocardial dysfunction [140]. A catecholamine storm has been described after brain death, correlated with the rate of intracranial pressure rise [141]. Hearts, transplanted after brain death, commonly displayed ventricular dysfunction [142]. Furthermore, it is well-recognized that acute emotional stress leads to heart failure and abnormalities of contraction [143]. Interestingly, the condition Takutsubo cardiomyopathy, additionally demonstrates the association between the heart and brain. His condition frequently includes partial hypokinesis/dyskinesia and an apical ballooning [144], which have been described in the context of pheochromocytoma [145]. Patients with pheochromocytoma frequently displayed reduced systolic strain rates in tissue doppler ECHO and 75% experience intraoperative collapse [146]. The apical ballooning in Takutsubo cardiomyopathy can be explained by higher β-adrenoreceptor density at the apical area of the heart (demonstrated in dogs but not humans) [147]. This form of stress-related cardiomyopathy is associated with ECG abnormalities and includes T-wave inversions that are not readily differentiable from an acute myocardial infarction [148]. In accordance with these observations, a recently published report related psychosocial stress to structural alterations in the heart structure, including Cx43 translocation and alterations in the z-disc located proteins actinin and desmin [105].

In the clinics, catecholamines are used to increase blood pressure and cardiac output with the aim to improve regional organ perfusion in shock. However, some catecholamines have been shown to have negative effects on microcirculation. Norepinephrine has been demonstrated to reduce microcirculation [149]. Furthermore, other adverse events during catecholamine therapy such as tachycardia, arrhythmias, and even immunologic and metabolic effects have been described. High catecholamine levels lead to vascular spasm and represent a potential source of free radicals, leading to impaired myocyte viability via calcium increase [142,150]. The catecholamine axis, aside from parasympathetic nerve function, is the most important connection between the brain and heart and may be responsible for cardiac damage after TBI. The catecholamine axis, aside from parasympathetic nerve function, is the most important connection between the brain and heart and may be responsible for cardiac damage after TBI. The role of catecholamines in the development of cardiac damage was also reported in the case of burn injury: after severe burn injury endogenous catecholamine levels are elevated and a persistent β-adrenergic receptor stimulation induces calcium dyshomeostasis, triggering the increase of inflammatory mediators, the decrease of SERCA2 and ryanodin receptor expression via MAPK increase and Akt phosphorylation, the increase of nitric oxide levels and the increase of cardiomyocyte apoptosis, resulting in cardiac dysfunction and damage [130]. Furthermore, there is an association between therapeutic catecholamine levels and the systemic increase of troponin in multiple trauma patients [9]. Moreover, post-traumatic elevation of catecholamines is associated with biomarkers of tissue and endothelial damage (Syndescan-1), glycocalyx degradation, coagulopathy including hyperfibrinolysis as well as independently predicts mortality in trauma patients [151,152]. The cardiotoxicity of large therapeutic doses of β-adrenergic receptor agonists is also described in the developing heart by inducing malformations of the cardiovascular system [153]. In the adult heart, mechanisms of cardiotoxicity of ‘therapeutic’ catecholamines are well-investigated. For example, catecholamines have been associated with necrotic/ischemic lesions of the myocardium by strong positive chronotropic and inotropic myocardial stimulation, as well as a by a reduced blood supply of the heart in rats [154]. Furthermore, early studies showed that catecholamines induce the influx of Ca^2+^ from extracellular space into cardiac cells, leading to calcium overload [155]. All in all, endogenous and exogenous catecholamines are important mediators of secondary cardiac damage.

Another relevant related condition is Takutsubo neurogenic stress-related cardiomyopathy due to subarachnoid hemorrhage (SAH), which is associated with fatal arrythmias and an increased risk for cerebral vasospasm [156]. Within the first days, 28% of SAH patients developed a regional left ventricular dysfunction, whereas 15% displayed global dysfunction with an ejection fraction < 50% [157]. The observed segmental dysfunction was not correlated with coronary artery distribution and was reversible in most cases [157]. The associated hypokinesia was observed more frequently in the basal and midventricular myocardium and is therefore referred to as the inverse Takutsubo [156,157]. In addition, troponin I levels were elevated in patients after SAH [158]. Neurogenic stress-related cardiomyopathy was also reported after stroke or TBI [148]. After stroke, prolonged ECG monitoring is recommended, particularly in patients with insular cortex involvement or high troponin T, because of an association with AF [159]. AF is an example for the association between heart disease and brain injury. In summary, published evidence supports that the association between the brain and heart is through sympatric/catecholamine interactions. Another described case of enhanced sympathetic tone inducing endogenous catecholamine stimulation of the myocardium is the phenomenon of voodoo death described earlier by Cannon et al. [160]. In summary the connection between brain and heart is not a new phenomenon and was extensively discussed previously and investigated in detail for example in post-mortem studies.

Another critical potential cause of indirect cardiac trauma is bone fractures. Twenty five percent of elderly patients developed a major adverse cardiac event (all-cause deaths, heart failure, new-onset AF, myocardial infarction (MI), and cardiovascular rehospitalization) within 90 days after hip fracture surgery [161]. Furthermore, a correlation between troponin, N-terminal B-type natriuretic peptide, and age-related reduced ejection fraction and major adverse cardiac events was described [161]. The incidence of coronary heart disease is higher in patients after hip fracture compared to healthy controls [162,163]. Following hip fracture, the highest risk for cardiac event is observed within the first year after hip fracture [162]. Whereas after blunt chest trauma, an eight-fold increase of MI risk was observed, pelvic or abdominal trauma in patients increased the risk of MI six-fold [164]. The development of a trauma-induced secondary cardiac injury was associated with the hyper-acute elevation of inflammatory cytokines including TNF, IL-6, IL-1β, and IL-8 [164,165]. Following hip fracture, underweight (<18.5 kg/m^2^) elderly (mean age 84.2 years) patients had a significantly higher risk for developing myocardial infarction and arrhythmias, compared to patients with normal body mass index (18.5–24.9 kg/m^2^) [166]. TNF is known to be elevated after fracture [167,168] and to be cardio-depressive as previously described [169,170]. TNF induced apoptosis in CMs and led to cardiac dysfunction in different models [169,171,172]. Furthermore, the presence of TNF led to elevations of ROS, troponin I release, and histone appearance in the supernatant of human CMs [165].

**Figure 1:**
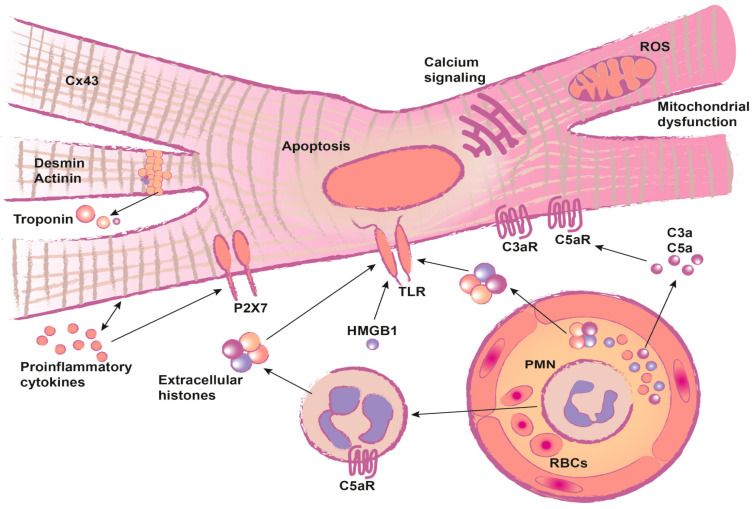
Molecular mechanism of post-traumatic cardiac dysfunction.

Further systemic, experimental studies are needed to clarify the dimensions and mechanisms (summarized in Figure 1) of cardiac injury after fracture.

### 2.4. Treatment Strategies

Most of the therapeutic approaches which might influence the cardiac damage after trauma are only experimental and without any application in the clinical field. Clinical trials are very limited and therefore clinical data are currently inconclusive. Therefore, additional well-designed human studies are needed to evaluate the real therapeutic potential.

Following hemorrhagic shock with high HMGB-1, Zhou et al. introduced anti-HMGB anti-HMGB-1 antibody as a therapeutic option (2015). They observed a decrease in cardiac enzyme levels, reduced ATP loss, protection of cardiac tissue, lower inflammatory mediators including TNF and IL-1β, reduction in apoptotic response, and a decreased TLR-4 expression compared to animals with hemorrhagic shock without therapy [173]. A further therapeutic option might be the Cytosorb^®^300 hemadsorption filter system, which was described to be useful for the elimination of DAMPs including HMGB-1 and histones, complement factors and cytokines [174,175]. Histone levels were experimentally reduced by systemic application of anti-histone antibody [60,176]. The use of histone neutralizing antibodies reduced the mortality after LPS or TNF infusion [177].

Another described novel therapeutic option is quercetin. Post-traumatic treatment with quercetin reduced CM apoptosis by suppressing TNF release, reducing ROS production, and ameliorating calcium increase in the CM [178]. Topisetron could also be an option, because of its ability to improve cardiac function and reduce apoptosis, MPO activity, and local IL-6 concentrations in the heart [179]. Treatment of trauma hemorrhage with topisetron attenuated apoptosis and further prevented the functional impairment of the heart as measured by cardiac output, +dp/dt max, −dp/dt max, and the mean arterial pressure [179]. The improvement of cardiac function due topisetron has been explained by its effect on Akt phosphorylation reduction [179].

Another option to reduce cardiac amount of TNF, IL-6, and NFkB after trauma-induced hemorrhagic shock is treatment with glucosamine during resuscitation. Glucosamine was found to improve cardiac function by increasing O-GlcNacylation and suppressing the NF-kB pathway [180]. All in all, there are some experimental approaches based on the molecular mechanisms of cardiac dysfunction which might be interesting in future. However, well-designed human studies are needed to evaluate the real therapeutic potential of these agents.

## 3. Conclusions

It is critical to understand the molecular mechanisms of both direct and indirect trauma. While cardiac damage is expected after direct injury of the chest or in patients with multiple trauma, secondary cardiac damage should be expected in clinical situations with increased inflammation and stress, including on fracture, TBI or burn injury. The mechanisms of post-traumatic cardiac dysfunction are multifactorial and include activation of the complement system, local and systemic inflammation, oxidative/nitrosative stress, apoptosis, modification of structural proteins, gap junction alteration, and changes in calcium handling.

## Data Availability

Data available on request due to restrictions, e.g., privacy or ethical.

## Supplementary Materials

The supplementary materials are available online at https://www.mdpi.com/1422-0067/22/2/737/s1.

## Abbreviations

AF	atrial fibrillation
AIS	abbreviated injury scale
Akt	protein kinase B
C5a/R	complement factor 5a/receptor
CINC	cytokine-induced neutrophil chemoattractant
CM	cardiomyocyte
DAMPs	danger-associated molecular patterns
ECG	electrocardiogram
ECHO	echocardiography
EF	ejection fraction
ERK	extracellular signal-regulated kinase
GLUT	glucose transporter
HF	heart failure
HFABP	heart type fatty acid binding protein
HMGB-1	high mobility group box-1 protein
ICAM	intercellular adhesion molecule
ICU	intensive care unit
IL	interleukin
iNOs	inducible nitric oxide synthase
ISS	injury severity score
JAM	junctional adhesion molecule
MAPK	mitogen-activated protein kinase
MI	myocardial infarction
MMP	matrix metalloprotease
MPO	myeloperoxidase
NADPH	nicotinamide adenine dinucleotide phosphate
NFkB	nuclear factor kappa-light-chain-enhancer of activated B cells
NO	nitric oxide
NT-proBNP	N-terminal B-type natriuretic peptide
PAMPs	pathogen-associated molecular patterns
PI3K	phosphoinositide 3-kinase
ROS	reactive oxygen species
SAH	subarachnoid hemorrhage
SAPS II	simplified acute physiology score
SERCA	sarco-/endoplasmic reticulum calcium ATPase
SIRS	systemic inflammatory response syndrome
TBI	traumatic brain injury
TISCI	trauma-induced secondary cardiac injury
TLR	toll-like receptor
TNF	tumor necrosis factor
TnI/T	troponin I/T
TUNEL	terminal deoxynucleatidyl transferase dUTP nick end labeling
ZO-1	Zonula occludens-1
