# Supplementary material for: Trauma, a Matter of the Heart—Molecular Mechanism of Post-Traumatic Cardiac Dysfunction
Source: Int J Mol Sci. 2021 Jan 13;22(2):737. doi: 10.3390/ijms22020737 (PMC7828409; doi:10.3390/ijms22020737)
Supplement: Supplementary file 1 [file ijms-22-00737-s001.pdf]

Supplementary Materials

Trauma, a Matter of the Heart—Molecular Mechanism of Post-Traumatic Cardiac Dysfunction

Birte Weber [1], Ina Lackner [1], Florian Gebhard [1], Theodore Miclau [2] and Miriam Kalbitz [1,*]

[1] Department of Traumatology, Hand-, Plastic-, and Reconstructive Surgery, Center of Surgery, University of Ulm, 86081 Ulm, Germany; birte.weber@uni-ulm.de (B.W.); ina.lackner@uni-ulm.de (I.L.); florian.gebhard@uniklinik-ulm.de (F.G.)

[2] Orthopaedic Trauma Institute, Department of Orthopaedic Surgery, University of California, 2550 23rd Street, San Francisco, CA 94110, USA; Theodore.Miclau@ucsf.edu

* Correspondence: miriam.kalbitz@uniklinik-ulm.de

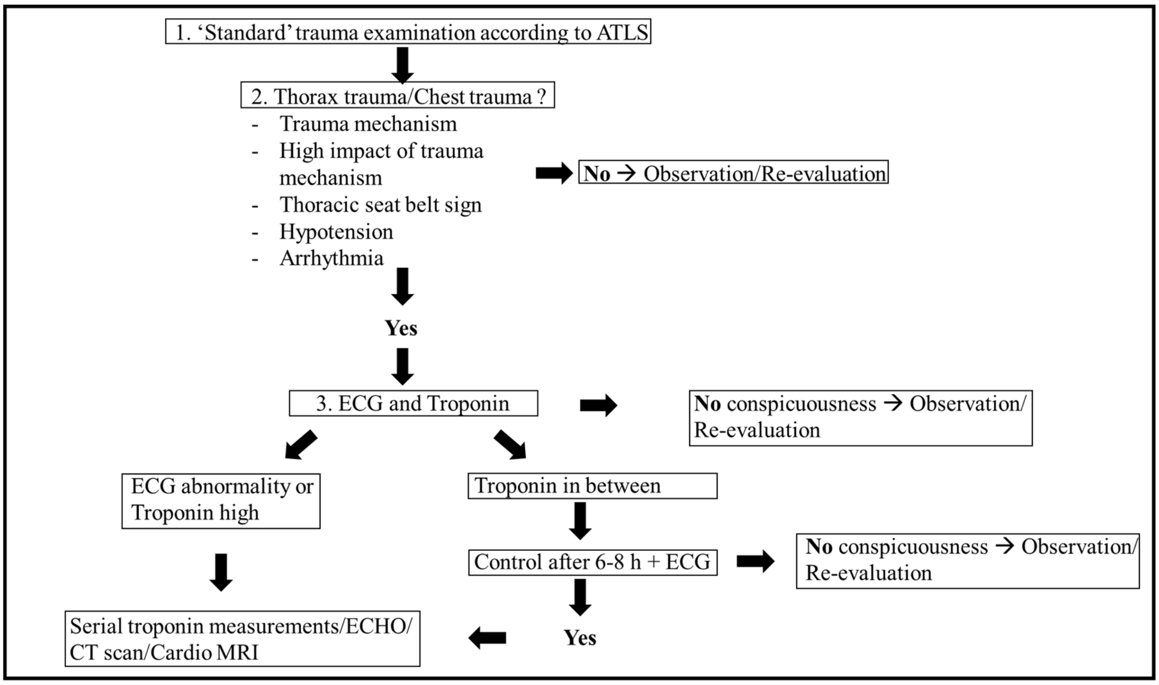

Supplementary Figure S1. Diagnostic algorithm for cardiac damage.
